# Reproductive Hormone Levels in Men Exposed to Persistent Organohalogen Pollutants: A Study of Inuit and Three European Cohorts

**DOI:** 10.1289/ehp.8935

**Published:** 2006-06-08

**Authors:** Aleksander Giwercman, Anna Rignell-Hydbom, Gunnar Toft, Lars Rylander, Lars Hagmar, Christian Lindh, Henning S. Pedersen, Jan K. Ludwicki, Vladimir Lesovoy, Maryna Shvets, Marcello Spano, Gian Carlo Manicardi, Davide Bizzaro, Eva C. Bonefeld-Jorgensen, Jens Peter Bonde, INUENDO

**Affiliations:** 1 Molecular Reproductive Medicine Research Unit, Department of Clinical Sciences, Malmö University Hospital, Lund University, Malmö, Sweden; 2 Department of Occupational and Environmental Medicine, Lund University Hospital, Lund, Sweden; 3 Department of Occupational Medicine, Aarhus University Hospital, Aarhus, Denmark; 4 Centre for Arctic Environmental Medicine, Nuuk, Greenland; 5 Department of Environmental Toxicology, National Institute of Hygiene, Warsaw, Poland; 6 Regional Clinical Center of Urology and Nephrology, Kharkiv State Medical University, Kharkiv, Ukraine; 7 Section of Toxicology and Biomedical Sciences, BIOTEC-MED, ENEA Casaccia Research Centre, Rome, Italy; 8 University of Modena and Reggio Emilia, Reggio Emilia, Italy; 9 Istituto di Biologia e Genetica, Università Politecnica delle Marche, Ancona, Italy; 10 Institute of Public Health, Department of Occupational and Environmental Medicine, University of Aarhus, Aarhus, Denmark

**Keywords:** CB-153, endocrine disruptors, estradiol, follicle-stimulating hormone, FSH, inhibin B, LH, luteinizing hormone, persistent organohalogen pollutants, *p,p′*-DDE, reproductive hormones, sex hormone-binding globulin, SHBG, testosterone

## Abstract

**Objective:**

Persistent organohalogen pollutant (POP) exposure may have a negative impact on reproductive function. The objective of this study was to assess the impact of POP exposure on the male hypothalamo–pituitary–gonadal axis.

**Participants:**

Participants included 184 Swedish fishermen and spouses of pregnant women from Greenland (*n* = 258), Warsaw, Poland (*n* = 113), and Kharkiv, Ukraine (*n* = 194).

**Evaluations/measurements:**

Serum levels of 2,2′,4,4′,5,5′-hexachlorobiphenyl (CB-153) and dichlorodiphenyl dichloroethene (*p*,*p*′-DDE) were determined in the four populations, showing different exposure patterns: Swedish fishermen, high CB-153/low *p*,*p*′-DDE; Greenland, high CB-153/high *p*,*p*′-DDE; Warsaw, low CB-153/moderate *p*,*p*′-DDE; Kharkiv, low CB-153/high *p*,*p*′-DDE. Serum was also analyzed for testosterone, estradiol, sex hormone-binding globulin (SHBG), inhibin B, luteinizing hormone (LH), and follicle-stimulating hormone (FSH). Free testosterone levels were calculated based on testosterone and SHBG.

**Results:**

We found significant center-to-center variations in the associations between exposure and the outcomes. The most pronounced effects were observed in Kharkiv, where statistically significant positive associations were found between the levels of both CB-153 and *p*,*p*′-DDE and SHBG, as well as LH. In Greenland, there was a positive association between CB-153 exposure and LH. In the pooled data set from all four centers, there was positive association between *p*,*p*′-DDE and FSH levels [β = 1.1 IU/L; 95% confidence interval (CI), 1.0–1.1 IU/L]. The association between CB-153 levels and SHBG was of borderline statistical significance (β = 0.90 nmol/L; 95% CI, −0.04 to 1.9 nmol/L).

**Conclusions:**

Gonadotropin levels and SHBG seem to be affected by POP exposure, but the pattern of endocrine response is the subject of considerable geographic variation.

During recent years, it has been widely discussed whether environmental chemicals mimicking or inhibiting the action of endogenous hormones, the so-called endocrine disruptors (EDs), have an adverse effect on male reproductive function (Skakkebæk et al. 2001; Toppari et al. 1996). This debate followed reports indicating negative secular trends in sperm counts and concomitant increases in the incidence of testicular cancer as well as congenital abnormalities of male genitalia, such as cryptorchidism and hypospadias (Giwercman et al. 1993). These abnormalities, together with some forms of male infertility, constitute the so-called testicular dysgenesis syndrome (TDS). Furthermore, it has been suggested that TDS is due to a hormonal imbalance in the male reproductive system caused by fetal exposure to EDs (Skakkebæk et al. 2001).

Compounds with potential ED effects, including persistent organohalogen pollutants (POPs), such as polychlorinated dibenzo-furans, polychlorinated dibenzo-*p*-dioxins, polychlorinated biphenyls (PCBs), dichloro-diphenyltrichloroethane (DDT), and dichloro-diphenyldichloroethene (*p*,*p*′-DDE; the most stable daughter compound of DDT), are ubiquitous environmental contaminants. These compounds are highly persistent, which results in bioaccumulation and biomagnification in the food chain. Measurable levels of PCBs and *p*,*p*′-DDE are found in a large proportion of the general population (Longnecker et al. 1997). Some of these chemicals can disrupt multiple endocrine pathways and induce a wide range of toxic responses (Toppari et al. 1996). A variety of studies have demonstrated their estrogenic, antiestrogenic, and androgen-interfering properties (Danzo 1997; Kelce et al. 1995). Furthermore, some of the PCBs have dioxin-like activity and therefore, through binding to the aryl hydrocarbon receptor (AhR) (Pocar et al. 2005), indirectly modify sex steroid action.

Provided that POPs can act as EDs, one should expect these compounds to interfere with the normal hypothalamo–pituitary–gonadal axis. However, the data regarding such associations are relatively limited, and the overall picture of the effect of exposure on hormone levels is not uniform.

It is not feasible to analyze all of the several hundred POP compounds that might be detected in human serum. Therefore, reliable proxy markers of exposure need to be used. The PCB congener 2,2′,4,4′5,5′-hexachloro-biphenyl (CB-153), found in relatively high concentrations in human serum, has been selected as a biomarker for POP exposure because of its very high correlation with the total PCB concentration (Glynn et al. 2000; Grimvall et al. 1997), the 2,3,7,8-tetra-chlorodibenzo-*p*-dioxin (TCDD) equivalent (TEQ) from PCBs, and the total POP-derived TEQ (Gladen et al. 1999a) in Swedish and North American populations. Likewise, the major DDT metabolite *p*,*p*′-DDE, an anti-androgenic compound, is another good indicator of POP exposure. Previous studies from Greenland, Sweden, Poland, and Ukraine indicate that the exposure for *p*,*p*′-DDE is still considerable (Czaja et al. 1997; Deutch and Hansen 2000; Gladen et al. 1999b; Sjödin et al. 2002).

As a part of a European Union–supported action, the impact of POP exposure on different aspects of male reproductive function was investigated in three European populations and among Greenland Inuit (Biopersistent Organochlorines in Diet and Human Fertility: Epidemiologic Studies of Time to Pregnancy and Semen Quality in Inuit and European Populations; INUENDO 2006). The aim of the present study was to assess the possible association between levels of CB-153 and *p*,*p*′-DDE in serum and reproductive hormones in males. Our hypothesis was that, provided that POPs act as EDs, the levels of the two exposure markers should to some degree correlate with concentrations of markers of testicular [testosterone, estradiol (E_2_), inhibin B] and/or pituitary [follicle-stimulating hormone (FSH) and luteinizing hormone (LH)] function. The action of POPs on the hypothalamo–pituitary–gonadal axis might be primarily exerted through the testis or via the hypothalamus/hypophysis. Because these chemicals can possess a multitude of endocrine effects, we did not, *a priori*, hypothesize which type of hormonal changes would be expected.

## Materials and Methods

### Study populations

The present study is part of a European study on fertility (INUENDO) using a uniform protocol for data collection in Greenland, Sweden, Ukraine, and Poland. The details of recruitment of study subjects have been described previously (Toft et al. 2005). In Greenland, Warsaw, Poland, and Kharkiv, Ukraine, the men were recruited for semen and blood sampling through their female partners attending the antenatal care clinics.

Briefly, the population from Greenland represents a population of Inuit with average exposure to high concentrations of POPs, because these compounds are bioaccumulated in sea mammals, which constitute a large part of their traditional food. In Ukraine, a recent study indicated current or recent exposure to DDT; in other parts of Europe, DDT has been banned and not used for about 30 years (Gladen et al. 1999b). In Poland, we included a population around the city of Warsaw, representing the exposure level in a large central European city. The concentrations of PCBs and DDE in breast milk samples from Warsaw are similar to those found in recent studies from other European regions (Czaja et al. 1997).

Swedish fishermen were recruited from a cross-sectional semen study (Rignell-Hydbom et al. 2004). East coast fishermen are highly exposed to POPs through fatty fish from the Baltic Sea. To secure a large variation in POP exposure, fishermen from the Swedish west coast were also included. The latter group has previously been shown to have POP concentrations at the same level as the general Swedish population and three times lower than the east coast fishermen (Svensson et al. 1995). The Swedish data have been reported separately (Rignell-Hydbom et al. 2004).

The following standard criteria were used for inclusion of men in the study: *a*) > 18 years of age, *b*) born in the country where the study was performed, and *c*) demonstrated fertility by having a pregnant or recently pregnant wife, except for the Swedish part of the study, where all fishermen were eligible if the first two criteria were fulfilled. However, 80% of the Swedish fishermen had fathered a child (Rignell-Hydbom et al. 2004).

In each center, the goal for recruitment was 200 men for the semen study; subjects who participated were as follows: in Greenland, 201 (79% participation rate); in Warsaw 198 (29%); in Kharkiv, 208 (8%); and in Sweden, 195 (8%). However, 105 men from Greenland, 85 from Warsaw, 14 from Kharkiv, and 11 from Sweden had to be excluded because of missing information on season and time of day when the blood sample was drawn. Another 162 men from Greenland, who did not participate in the semen study but were recruited in a similar way for a time-to-pregnancy (TTP) survey, were included in the analysis, giving a final total study group of 749 subjects.

A nonparticipant analysis performed for Greenland and Warsaw did not show any difference in reproductive hormone levels between subjects who had been excluded and those who remained in the final study group. Moreover, the hormone levels in the 162 men from Greenland included from the TTP study were similar to those in the 96 who delivered semen samples.

The local ethics committees representing all participating populations approved the study, and all subjects signed informed consents.

### Collection of blood samples

Blood samples were drawn from a cubital vein into 10-mL vacuum tubes for serum collection without additives (Becton Dickinson, Maylan, France). After cooling to room temperature, the tubes were centrifuged at 4,000 × *g* for 15 min. Serum was transferred with ethano-rinsed Pasteur pipettes to ethanol-rinsed brown glass bottles (Termometerfabriken, Gothenburgh, Sweden). A piece of aluminum foil was placed on top of the bottles, which were then sealed. After a maximum of 4 days in the refrigerator, sera were stored at −20°C until shipment on dry ice and kept frozen until analysis.

### Measures of exposure

Serum concentrations of CB-153 and *p*,*p*′-DDE were analyzed by gas chromatography/mass spectrometry after solid-phase extraction (Richthoff et al. 2003). POP levels were adjusted for serum lipids determined by enzymatic methods.

### Hormone analyses

Measurements of FSH, LH, and E_2_ were made using the UniCel DxI 800 Beckman Access Immunoassay system (Chaska, MN, USA). The lower limits of detection (LODs) for the assays were 0.2 IU/L, 0.2 IU/L, and 8.0 pmol/L, respectively. The total assay coefficients of variation (CVs) were as follows: for FSH, 3.5% at 5.5 IU/L and 4.1% at 23.6 IU/L; for LH, 5.2% at 4.0 IU/L and 2.3% at 19.3 IU/L; and for E_2_, 17.4% at 44 pmol/L and 6.7% at 303 pmol/L. Serum testosterone levels were measured by means of a competitive immunoassay (Access; Beckman Coulter Inc., Fullerton, CA, USA) with an LOD of 0.35 nmol/L and a total assay CV of 2.8% at 2.9 nmol/L and 3.2% at 8.1 nmol/L. Sex hormone-binding globulin (SHBG) concentrations were measured using a fluoro-immunoassay (Immulite 2000; Diagnostic Products Corporation, Los Angeles, CA, USA). The LOD was 0.02 nmol/L, and the total assay CV was 3.7% at 29 nmol/L and 6.7% at 85 nmol/L. Values of free testosterone (fT) were calculated from total serum testosterone and SHBG concentrations using the formula by Vermeulen et al. (1999).

Inhibin B levels were assessed using a specific immunometric method as previously described (Groome et al. 1996), with an LOD of 15 ng/L and intraassay and total assay CVs < 7%.

All assays were performed at Malmö University Hospital after the completion of sample collection. Interassay variation was minimized by analyzing samples from the same center in the same batch.

### Statistical analysis

The association between exposure and hormone levels (fT, E_2_, SHBG, inhibin B, FSH, LH) was tested with linear regression. Because the fT rather that the total fraction of testosterone is considered as being biologically active, only the SHBG-adjusted fT (Vermeulen et al. 1999) and not total testosterone levels are given. To achieve normal distribution of the residuals, both exposure parameters and concentrations of LH and FSH were transformed by the natural logarithm. The log-transformed values are presented in the tables showing linear regression results, whereas back-transformed values are given in the text.

The list of potential confounders included body mass index (BMI) (< 20, 20 to ≤ 25, > 25 to ≤ 30, > 30), smoking (yes/no), alcohol consumption (≤ 21 or > 21 alcohol drinks per week), season of sampling (summer, autumn, winter, spring), time of sample collection (before 1200 hr or 1200 hr or later), and age (years, as a continuous variable). Variables were included in the basic model one by one and were kept for further analysis only if the risk estimate was changed at least 10%. The potential confounders were added to the model in a stepwise order according to their effect on the risk estimate but were kept in the model only if the exclusion changed risk estimate at least 5%. The characteristics of the study populations with respect to exposure, outcome variables, and potential confounders are given in Table 1.

Hormone levels were compared in the three cohorts of partners of pregnant females (Greenland, Warsaw, and Kharkiv), with men from Greenland serving as a reference population.

Apart from testing the exposure levels as continuous variables, the CB-153 and *p*,*p*′-DDE variables were analyzed as five arbitrarily categorized groups (0–50, 51–100, 101–200, 201–400, and ≥ 401 ng/g lipid for CB-153; 0–50, 51–100, 101–200, 201–400, and ≥ 401 ng/g lipid for *p*,*p*′-DDE) (Table 2). The categorized variables were entered as dummy variables in the regression models. For each hormone parameter combination, we tested the difference between the highest and lowest exposure groups with sufficient numbers of subjects for performing statistical analysis (the subjects within the groups were unevenly distributed between the centers because of different exposure profiles).

Because the CB-153 and *p*,*p*′-DDE serum levels, especially in Inuit and Swedish fishermen, were highly correlated (*r* = 0.93 and 0.79, respectively) (Jönsson et al. 2005), both variables were not taken into the models simultaneously.

Initially, all analyses were stratified by the four study groups. Thereafter, we performed a statistical test for heterogeneity of risk including significance test for interaction terms in multiple linear regression models. If the interaction between the exposure and the region in relation to the given outcome was not statistically significant, we estimated the strength of the associations based on data aggregated from several regions, including study group as a covariate in the models.

All analyses were carried out using SPSS software (SPSS for Windows, version 11.0; SPSS Inc., Chicago, IL, USA).

## Results

### Between-center variation in hormone levels

When comparing the three cohorts of partners to pregnant women, we observed statistically significant differences in serum levels for all hormones except LH. With Greenland as reference, men from Warsaw presented with lower fT and SHBG levels and those from Kharkiv with higher fT levels. The E_2_ concentration was higher in both Warsaw and in Kharkiv; for inhibin B and FSH, the only difference was lower levels in Warsaw. The results are summarized in Table 3.

The results of linear regression analyses for continuous exposure variables are given in Tables 4 and 5. Below, we present a summary of the results according to the sample collection center.

### Greenland

We found a weak but statistically significant positive association [β = 0.011 nmol/L; 95% confidence interval (CI), 0.0004–0.024 nmol/L; *p* = 0.04] between *p*,*p*′-DDE levels and fT (Table 5). The highest exposure group for CB-153 presented with significantly higher LH levels compared with the lowest group (mean difference, 1.4 IU/L; 95% CI, 1.1–1.7 IU/L; *p* = 0.02). The highest *p*,*p*′-DDE group presented with significantly higher inhibin B levels (mean difference, 35 ng/L; 95% CI, 1.5–69 ng/L; *p* = 0.04) than did the lowest group.

### Warsaw

No statistically significant associations were found in the linear regression analysis using continuous exposure levels. In the analysis using categorized exposure, fT levels were significantly lower in the third highest CB-153 group (the highest in Poland) (mean difference, −0.07 nmol/L; 95% CI, −0.1 to −0.01 nmol/L; *p* = 0.01) compared with the lowest group. The highest *p*,*p*′-DDE group did not differ from the lowest group for any of the hormone parameters tested.

### Swedish fishermen

We found no statistically significant associations in Swedish fisher-man, using either continuous or categorized exposure variables.

### Kharkiv

**Figure 1:**
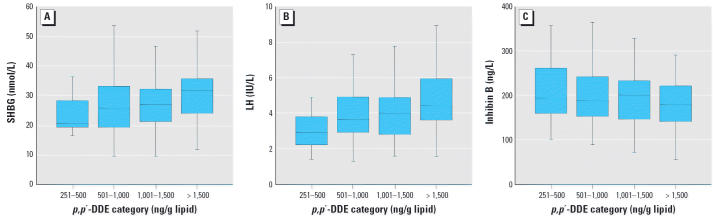
Box plots showing the association between *p*,*p*′-DDE exposure levels (ng/g lipid) and serum concentrations of SHBG (*A*), LH (*B*), and inhibin B (*C*) among the 194 men from Kharkiv. Values shown are median (lines), interquartile range (boxes), and range, excluding extremes and outliers (whiskers). The subjects were allocated into five groups according to the exposure level for all four study groups; however, no Kharkiv subjects were in the lowest exposure group (≤ 250 ng/g lipid).

A statistically significant positive association was found between CB-153 and SHBG (β = 3.6 nmol/L; 95% CI, 1.7–5.5 nmol/L; *p* < 0.0005) as well as LH (β = 1.1 IU/L; 95% CI, 1.0–1.2 IU/L; *p* = 0.04) (Table 4). For *p*,*p*′-DDE, we observed significant associations with SHBG (β = 3.6 nmol/L; 95% CI, 1.3–5.9 nmol/L; *p* = 0.003), LH (β = 1.3 IU/L; 95% CI, 1.1–1.4 IU/L; *p* < 0.0005), and inhibin B (β = −17.0 ng/L; 95% CI, −33.6 to −0.473 ng/L; *p* = 0.04). We also found an association between SHBG concentration and CB-153 exposure when exposure groups were compared (Figure 1). The third highest exposure group (only one subject was categorized into the two highest groups) presented with higher SHBG (mean difference, 7.5 nmol/L; 95% CI, 2.8–12 nmol/L; *p* = 0.002) compared with the lowest exposure category. Similarly, the highest *p*,*p*′-DDE group presented with significantly higher levels of SHBG (mean difference, 6.8 nmol/L; 95% CI, 2.3–11 nmol/L; *p* = 0.01) and LH (mean difference, 1.6 IU/L; 95% CI, 1.2–2.0 IU/L; *p* < 0.0005) and lower inhibin B concentrations (mean difference, −40 ng/L; 95% CI, −76 to −3.3 ng/L; *p* = 0.03) compared with the second lowest group (no subjects in the lowest group).

### Pooled data

Heterogeneity testing showed that data from the four study groups could be pooled for all exposure–outcome combinations, except for LH versus the continuous exposure variables. The only statistically significant finding was a positive association between *p*,*p*′-DDE and FSH levels (β = 1.1 IU/L; 95% CI, 1.0–1.1 IU/L; *p* = 0.03). The association between CB-153 levels and SHBG was borderline statistically significant (β = 0.90 nmol/L; 95% CI, −0.04 to 1.9 nmol/L; *p* = 0.06).

## Discussion

This study, based on four cohorts of men exposed to different levels of PCBs and *p*,*p*′-DDE, provided some but not unequivocal evidence of a dose-dependent impact of POP exposure on levels of male reproductive hormones. However, the association between exposure and the outcome differed significantly among the four populations. The most pronounced effects were seen in Kharkiv and Greenland: the two populations were characterized by high *p*,*p*′-DDE levels, and Greenland even by high CB-153 exposure. In Kharkiv, the CB-153 and *p*,*p*′-DDE exposures were positively associated with LH and SHBG levels. In Greenland, the same was true for association between CB-153 and LH, although because of a high correlation between levels of the two POP markers in this region (Jönsson et al. 2005), the effect of these two compounds cannot be discriminated. In both locations, the highest and lowest *p*,*p*′-DDE exposure groups differed with regard to inhibin B levels, although the association was positive in Greenland and negative in Kharkiv. In the pooled data set, the only statistically significant association was between *p*,*p*′-DDE and FSH; the association between CB-153 and SHBG was borderline significant.

Although the magnitude of exposure-related hormonal changes and also their direction differed among the four groups of men, such discrepancy is plausible. The four study groups represent populations rather heterogeneous with respect to their exposure profiles. Apart from the differences regarding the CB-153:*p*,*p*′-DDE ratio and the absolute serum levels of these two chemicals in the four populations studied, one cannot exclude that the pattern of exposure to other POPs also might be subject to significant geographic variation.

It seems likely that differing sex-hormone–mimicking effects of the various POPs, as shown *in vitro* (Bonefeld-Jorgensen et al. 2001), might contribute to the diverging effects regarding the levels of reproductive hormones. Indeed, *p*,*p*′-DDE exhibits an antiandrogenic effect, and PCBs may possess different types of sex hormone agonistic and antagonistic activity. Furthermore, some PCBs were shown to activate the AhR, which previously was shown to affect the action of androgen as well as estrogen receptors in prostate cells (Kizu et al. 2003; Pocar et al. 2005). Whether such interaction also can exist in other organs is not yet known. Thus, the sum ED effect of POP exposure depends not only on the levels of the two markers CB-153 and *p*,*p*′-DDE but also on the total panorama of chemicals to which the subject has been exposed.

Other factors contributing to the inter-center difference should also be considered. The Inuits represent an ethnic group different from the three European Caucasian populations and might, therefore, have differing genetically determined sensitivity to adverse effects of POPs. In Sweden and in Greenland, the source of exposure was consumption of seafood, whereas the source of POPs was unclear in Warsaw and in Kharkiv. Furthermore, in three of the centers, the men were selected as proven fertile men, whereas the inclusion of Swedish fishermen was not based on their fertility status. However, 80% of the participating fishermen had fathered a child. Comparison of the three cohorts of partners to pregnant women showed significant between-population differences regarding levels of all reproductive hormones except LH. Finally, major expected differences in overall dietary composition, rather than how this alters POP exposure, might be a more influential factor on hormone levels in the studied men. The POP exposure data might be partial markers for some of these differences (e.g., consumption of oily fish) and thus explain the lack of consistent effects between populations.

The overall INUENDO project has included a large number of reproductive end points, including standard semen parameters, levels of reproductive hormones, sperm chromatin integrity, and TTP. So far, the analysis of the available data (Axmon et al. 2006; Spano et al. 2005; Tiido et al. 2006; Toft et al. 2006) indicates significant cohort-to-cohort differences regarding the association between exposure levels and reproductive outcomes. The findings of the present study fit into this general pattern and support the above-mentioned factors as plausible explanations of this regional variation.

For each center, 24 comparisons were performed, and some of the statistically significant associations reported here might be chance findings because of mass significance, although this explanation does not seem likely in Kharkiv, where both CB-153 and *p*,*p*′-DDE exposure levels seemed to have an impact on several of the outcome variables. For both exposure markers, we found a positive association with SHBG concentration; this association was close to the level of statistical significance for the pooled data from the four centers. This observation is in accordance with a recent study performed on Swedish military conscripts (Richthoff et al. 2003). A positive association between POP exposure and SHBG was also found in a study of Swedish and Latvian men but was not significant after adjustment for age (Hagmar et al. 2001). In a study of 178 men, Persky et al. (2001) found a negative association between PCB exposure and SHBG-bound testosterone, without affecting levels of SHBG and fT. CB-153 was found to have an estrogen-like effect *in vitro* (Bonefeld-Jorgensen et al. 2001), whereas *p*,*p*′-DDE is known as an antiandrogen, both types of actions tending to stimulate liver synthesis of SHBG.

Both in Greenland and in Ukraine, we found that higher POP exposure levels were associated with an increase in LH, and in the pooled data set, *p*,*p*′-DDE levels were positively associated with FSH. These effects fit with the antiandrogenic effect of this compound. In rats, acute exposure to CB-153 caused a decrease in LH and FSH (Desaulniers et al. 1999). Occupational and accidental exposures to dioxins have been associated with a decrease in testosterone levels in adult men, whereas the findings with respect to gonadotropin levels were less uniform, with a positive association between exposure and FSH as well as LH in one study (Egeland et al. 1994), and no association in another (Henriksen et al. 1996). Among men highly exposed to DDT/DDE, decreased testosterone levels were observed (Ayotte et al. 2001; Dalvie et al. 2004; Martin et al. 2002), but no effects on gonadotropins were found in the two studies where these hormones were assessed (Ayotte et al. 2001; Dalvie et al. 2004). In less-exposed populations, no associations between *p*,*p*′-DDE and testosterone or gonadotropin levels were found (Cocco et al. 2004; Hagmar et al. 2001). Persky et al. (2001) found no effect of PCB on gonadotropins. In young Swedish males, there was a weak but significant negative correlation between CB-153 and fT in serum, but no effects on the gonadotropins (Richthoff et al. 2003). Studies on middle-age and elderly men have not revealed any association between PCB exposure and effects on the pituitary–gonadal axis (Hagmar et al. 2001). However, most of the above-cited studies were based on a relatively low number of subjects (< 200) and/or low-to-moderate exposures. In our study, the association between *p*,*p*′-DDE and FSH was statistically significant in the pooled data set but not in any of the regional cohorts.

Inhibin B is a Sertoli cell marker reflecting early stages of spermatogenesis. In Kharkiv, the levels of this hormone were decreased in the subjects with the highest *p*,*p*′-DDE exposure, whereas an opposite association was seen regarding the *p*,*p*′-DDE and CB-153 exposure in Greenland and in Warsaw, respectively. The levels of fT were positively associated with *p*,*p*′-DDE in Greenland but were not related to exposure in any other cohorts. In 37 Australian men exposed to low levels of TCDD, Johnson et al. (2001) found a negative correlation between the concentrations of TCDD and serum testosterone.

Thus, both the data presented here and in available literature give no clear picture with respect to the effect of POP exposure on levels of reproductive hormones. The effects seem in general rather limited and vary considerably from population to population. This phenomenon might at least partly be due to the causes suggested above: different exposure profiles, divergences in selection criteria and ethnicity, and chance findings due to multiple comparisons.

When evaluating the results of the present study, apart from the issue of multiple testing, potential biases need to be considered. Most men included in this study of hormone levels were those who agreed to deliver a semen sample for analysis. Except for the Inuits, the participation rate in the semen study, as in similar surveys, was rather low. Regarding the Swedish fishermen, the age distributions and the mean number of children were very similar among the participants and the nonparticipants (Rignell-Hydbom et al. 2004). In the three remaining cohorts, TTP did not differ between those who delivered semen for analysis and those who did not (Toft et al. 2005). Therefore, we do not consider selection bias to be of major concern. In the statistical analysis we have included all known potential confounders: BMI, smoking, alcohol consumption, season, time of blood sampling, and age. However, we cannot exclude that imperfect measurements of the confounders have caused some residual confounding.

In a study of juvenile alligators from Lake Apopka (Florida) and other American lakes, Guillette et al. (1999) found no correlation between PCB or DDE levels and sex steroid levels. The authors therefore concluded that the genital malformations rather frequently found among these animals are not associated with current levels of environmental contaminants but may be due to exposure during embryonic development. Similarly, the hypothesis of human TDS focuses on fetal exposure to chemicals with ED effects (Skakkebæk et al. 2001). The design of the present study does not allow estimation of the effect of fetal exposure in relation to levels of male reproductive hormones. However, two main conclusions of this study might have some relevance in the context of the suggestion of endocrine disruption as the cause of TDS. First, male reproductive hormone homeostasis—at least in adulthood—may to some degree be influenced by POP exposure, resulting in increasing gonadotropin and SHBG levels. Second, the pattern of endocrine response to the POP exposure seems to be subject of considerable geographic variation. This variation might contribute to the regional differences in semen quality and risk of testicular cancer and of genital congenital abnormalities reported previously.

## Figures and Tables

**Table 1:** Characteristics of the study populations with respect to exposure, outcome variables, and potential confounders.

Characteristic	Greenland (*n* = 258)	Warsaw (*n* = 113)	Swedish fishermen (*n* = 184)	Kharkiv (*n* = 194)	All (*n* = 749)
Exposure variables^a^					
PCB-153 (ng/g lipid)	190, 170 (5.1–5,500)	17, 18 (3.3–130)	190, 190 (40–150)	44, 47 (5.5–570)	90, 100 (3.3–5,500)
*p*,*p*′-DDE (ng/g lipid)	480, 500 (5.9–13,000)	530, 5,109 (200–2,100)	250, 190 (40–2,300)	1,100, 1,000 (320–12,000)	510, 530 (5.9–13,000)
Potential confounders					
Age (years)^a^	31, 30 (18–50)	31, 30 (20–46)	47, 48 (24–68)	27, 25 (19–45)	34, 32 (18–68)
Current smoking (%)	67	33	23	64	49
> 21 alcoholic drinks/week (%)	4.9	2.6	—	0	2.2
BMI < 20/20 to ≤ 25/> 25 to ≤ 30/> 30 (%)	3/41/40/14	2/40/45/12	0/32/53/15	6/59/33/3	3/44/42/11
Season of blood sampling, summer/autumn/winter/spring (%)	16/25/32/27	18/17/60/5	35/30/34/0	23/23/28/26	23/24/27/25
Time of blood sampling, before 1200 hr/after1200 hr (%)	12/88	5/95	39/61	87/13	50/50
Outcome variables^a^					
fT (nmol/L)	0.30, 0.30 (0.08–0.64)	0.29, 0.29 (0.12–0.51)	0.25, 0.23 (0.09–0.80)	0.39, 0.37 (0.11–0.84)	0.32, 0.30 (0.08–0.84)
SHBG (nmol/L)	29.5, 29.3 (11.1–61.9)	23.6, 21.7 (5.90–63.7)	31.4, 31.0 (6.80–71.1)	28.0, 27.0 (9.40–64.0)	28.7, 27.9 (5.90–71.1)
E_2_ (pmol/L)	64.2, 63.6 (26.8–264)	75.5, 68.6 (37.5–297)	70.2, 66.9 (25.4–155)	83.8, 78.2 (33.0–160)	72.4. 68.8 (25.4–297)
LH (IU/L)	3.88, 4.00 (1.40–13.2)	3.82, 3.70 (1.30–8.90)	3.97, 3.90 (1.50–19.6)	3.83, 4.00 (1.30–12.7)	3.88, 3.90 (1.30–19.6)
Inhibin B (ng/L)	173, 158 (13.0–470)	158, 153 (22.0–338)	190, 181 (2.00–433)	195, 188 (55.0–390)	180, 172 (2.00–470)
FSH (IU/L)	4.27, 4.20 (0.00–36.6)	3.48, 3.60 (0.70–16.7)	5.45, 5.10 (1.40–54.0)	3.72, 3.40 (1.00–21.3)	4.24, 4.16 (0.00–54.0)

a Values are mean, median (minimum–maximum).

**Table 2:** Numbers of subjects from the four study groups categorized by the distribution of CB-153 or *p,p*-DDE for the total group.

Exposure marker	Greenland (*n* = 258)	Warsaw (*n* = 113)	Swedish fishermen (*n* = 184)	Kharkiv (*n* = 194)
CB-153 (ng/g lipid)				
0–50	16	109	5	108
51–100	39	3	24	67
101–200	87	1	69	18
201–400	59	0	66	0
≥ 401	57	0	20	1
*p*,*p*′-DDE (ng/g lipid)				
0–250	58	7	100	0
251–500	71	47	52	17
501–1,000	68	50	24	80
1,001–1,500	33	6	6	46
≥ 1,500	28	3	2	51

**Table 3:** Adjusted mean (95% CI) hormone concentrations in the three cohorts of partners of pregnant women.

Hormone	Greenland (*n* = 258)	Warsaw (*n* = 113)	Kharkiv (*n* = 194)
fT (nmol/L)	0.34 (Ref)	0.30 (−0.07 to −0.01)	0.39 (0.02 to 0.07)
SHBG (nmol/L)	30.0 (Ref)	24.2 (−8.56 to −2.86)	27.7 (−4.79 to 0.27)
E_2_ (pmol/L)	67.0 (Ref)	78.8 (3.97 to 19.6)	87.5 (13.6 to 27.3)
lnLH (IU/L)	1.44 (Ref)	1.40 (−0.18 to 0.009)	1.38 (−0.17 to 0.06)
Inhibin B (ng/L)	178 (Ref)	149 (−49.9 to −7.75)	178 (−18.7 to 18.0)
lnFSH (IU/L)	1.43 (Ref)	1.26 (−0.33 to −0.001)	1.35 (−0.22 to 0.07)

Ref, reference population. The 95% CIs for the difference from the reference value (Greenland) are given. The values are adjusted for BMI, smoking, alcohol consumption, season, time of blood sampling, and age.

**Table 4:** Adjusted regression coefficients (β) for association between CB-153 lipid-adjusted levels and the outcome variables.

	Greenland (*n* = 258)		Warsaw (*n* = 113)		Swedish fishermen (*n* = 184)		Kharkiv (*n* = 194)		All (*n*= 749)	
Hormone	β	95% CI (β)	β	95% CI (β)	β	95% CI (β)	β	95% CI (β)	β	95% CI (β)
fT (nmol/L)	0.008^a^–^d^	−0.006 to 0.022	0.006^a^–^e^	−0.017 to 0.029	0.005^a^–^d^,^f^	−0.019 to 0.029	−0.005^a^,^d^,^f^	−0.029 to 0.018	0.003^a^–^f^	−0.007 to 0.012
SHBG (nmol/L)	−0.094^a^–^d^	−1.29 to 1.27	2.732^a^,^b^,^d^	−0.189 to 5.65	−1.345^a^,^b^,^d^	−4.47 to 1.78	3.598	1.74 to 5.46*	0.904^a^–^d^	−0.041 to 1.850
E_2_ (pmol/L)	−0.216^a^–^f^	−3.09 to 2.66	−3.388^b^,^e^	−12.3 to 5.57	−4.436^b^,^d^	−1.02 to 1.33	0.354^a^,^b^,^d^,^f^	−4.67 to 5.37	−1.27^a^–^f^	−3.58 to 1.05
lnLH (IU/L)	0.006^b^,^d^,^f^	−0.025 to 0.114	−0.115^d^	−0.231 to 0.0006	−0.070^b^,^d^	−0.191 to 0.051	0.092	0.005 to 0.175*	ND	ND
Inhibin B (ng/L)	6.16^a^,^b^,^d^	−3.93 to 16.3	−2.255^a^–^e^	−21.1 to 16.6	−6.385^a^,^b^,^d^	−28.1 to 12.7	−0.502^a^,^c^,^d^,^f^	−13.9 to 12.9	−0.880^a^–^f^	−7.43 to 5.67
lnFSH (IU/L)	0.030^a^–^d^,^f^	−0.048 to 0.097	0.097^a^,^b^,^d^	−0.261 to 0.067	1.07^a^,^b^,^d^,^f^	−0.876 to 1.16	0.042^d^	−0.065 to 0.145	0.021^a^–^f^	−0.029 to 0.072

ND, not done because of statistically significant heterogeneity.

Footnote letters correspond to the confounders that fulfilled the criteria for being included in the final model:

a BMI [low (< 20), normal (> 20 to ≤ 25), high (> 25 to ≤ 30), or obese (> 30)].

b Season (summer/autumn/winter/spring).

c Time of blood sampling (before 1200 hr/1200 hr or later).

d Age (years).

e Alcohol (≤ 21 alcohol drinks per week/> 21 alcohol drinks per week)

f Smoker (yes/no).

* Statistically significant association because 95% CI (β) does not include zero.

**Table 5:** The adjusted regression coefficients (β) for association between *p*,*p*′-DDE lipid-adjusted levels and the outcome variables.

	Greenland (*n* = 258)		Warsaw (*n* = 113)		Swedish fishermen (*n* = 184)		Kharkiv (*n* = 194)		All (*n*= 749)	
Hormone	β	95% CI (β)	β	95% CI (β)	β	95% CI (β)	β	95% CI (β)	β	95% CI (β)
fT (nmol/L)	0.011^a^–^c^	0.004 to 0.024*	−0.016^a^–^d^	−0.047 to 0.015	−0.007^a^–^e^	−0.026 to 0.012	0.008^a^,^c^,^d^,^f^	0.021 to 0.037*	0.006^a^–^f^	−0.003 to 0.015
SHBG (nmol/L)	−0.509^a^–^d^	−1.63 to 0.616	1.463^a^,^b^,^d^,^e^	−2.46 to 5.39	−0.951^a^,^c^,^d^	−3.52 to 1.61	3.57	1.27 to 5.88*	0.189^a^–^e^	−0.736 to 1.12
E_2_ (pmol/L)	0.516^a^–^f^	−1.99 to 3.03	−10.5^a^	−23.7 to 2.60	−0.355^a^–^d^	−0.324 to 0.364	3.23^d^	−2.81 to 9.28	0.090^a^–^f^	−2.17 to 2.35
lnLH (IU/L)	0.041^a^,^b^	−0.039 to 0.279	−0.019^a^–^e^	−0.194 to 0.156	0.014^a^,^b^,^d^,^f^	−0.085 to 0.113	0.227	0.124 to 0.324*	ND	ND
Inhibin B (ng/L)	9.01^d^,^f^	−0.123 to 18.9	−10.3^a^–^d^	−35.5 to 15.0	−5.325^a^–^d^	−20.0 to 0.476	−17.0^a^	−33.6 to −0.473*	−1.57^a^–^d^	−8.05 to 4.92
lnFSH (IU/L)	0.030^a^–^c^,^f^	−0.033 to 0.094	−0.012^a^,^b^,^d^,^e^,^f^	−0.211 to 0.236	0.122^a^,^c^,^d^	−0.046 to 0.290	0.115^c^	−0.015 to 0.245	0.056^a^–^f^	0.006 to 0.105*

ND, not done because of statistically significant heterogeneity.

Footnote letters correspond to the confounders that fulfilled the criteria for being included in the final model:

a Season (summer/autumn/winter/spring).

b Time of blood sampling (before 1200 hr/1200 hr or later).

c Age (years).

d BMI [low (< 20), normal (> 20 to ≤ 25), high (> 25 to ≤ 30), or obese (> 30)].

e Alcohol (≤ 21 alcohol drinks per week/> 21 alcohol drinks per week).

f Smoker ( (yes/no).

* Statistically significant association because 95% CI (β) does not include zero.

## References

[b1-ehp0114-001348] Axmon A, Thulstrup AM, Rignell-Hydbom A, Pedersen HS, Zvyezday V, Ludwicki JK (2006). Time to pregnancy as a function of male and female serum concentrations of 2,2′4,4′5,5′-hexachlorobiphenyl (CB-153) and 1,1-dichloro-2,2-bis (*p*-chlorophenyl)-ethylene (*p,p*′-DDE). Hum Reprod.

[b2-ehp0114-001348] Ayotte P, Giroux S, Dewailly E, Hernandez AM, Farias P, Danis R (2001). DDT spraying for malaria control and reproductive function in Mexican men. Epidemiology.

[b3-ehp0114-001348] Bonefeld-Jorgensen EC, Andersen HR, Rasmussen TH, Vinggaard AM (2001). Effect of highly bioaccumulated poly-chlorinated biphenyl congeners on estrogen and androgen receptor activity. Toxicology.

[b4-ehp0114-001348] Cocco P, Loviselli A, Fadda D, Ibba A, Melis M, Oppo A (2004). Serum sex hormones in men occupationally exposed to dichloro-diphenyltrichloro ethane (DDT) as young adults. J Endocrinol.

[b5-ehp0114-001348] Czaja K, Ludwicki JK, Goralczyk K, Strucinski P (1997). Organochlorine pesticides, HCB, and PCBs in human milk in Poland. Bull Environ Contam Toxicol.

[b6-ehp0114-001348] Dalvie MA, Myers JE, Lou TM, Dyer S, Robins TG, Omar S (2004). The hormonal effects of long-term DDT exposure on malaria vector-control workers in Limpopo Province, South Africa. Environ Res.

[b7-ehp0114-001348] Danzo BJ (1997). Environmental xenobiotics may disrupt normal endocrine function by interfering with the binding of physiological ligands to steroid receptors and binding proteins. Environ Health Perspect.

[b8-ehp0114-001348] Desaulniers D, Leingartner K, Wade M, Fintelman E, Yagminas A, Foster WG (1999). Effects of acute exposure to PCBs 126 and 153 on anterior pituitary and thyroid hormones and FSH iso-forms in adult Sprague Dawley male rats. Toxicol Sci.

[b9-ehp0114-001348] Deutch B, Hansen JC (2000). High human plasma levels of organochlorine compounds in Greenland. Regional differences and lifestyle effects. Dan Med Bull.

[b10-ehp0114-001348] Egeland GM, Sweeney MH, Fingerhut MA, Wille KK, Schnorr TM, Halperin WE (1994). Total serum testosterone and gonadotropins in workers exposed to dioxin. Am J Epidemiol.

[b11-ehp0114-001348] Giwercman A, Carlsen E, Keiding N, Skakkebæk NE (1993). Evidence for increasing incidence of abnormalities of the human testis: a review. Environ Health Perspect.

[b12-ehp0114-001348] Gladen BC, Longnecker MP, Schecter AJ (1999a). Correlations among polychlorinated biphenyls, dioxins, and furans in humans. Am J Ind Med.

[b13-ehp0114-001348] Gladen BC, Monaghan SC, Lukyanova EM, Hulchiy OP, Shkyryak-Nyzhnyk ZA, Sericano JL (1999b). Organochlorines in breast milk from two cities in Ukraine. Environ Health Perspect.

[b14-ehp0114-001348] Glynn AW, Wolk A, Aune M, Atuma S, Zettermark S, Maehle-Schmid M (2000). Serum concentrations of organochlorines in men: a search for markers of exposure. Sci Total Environ.

[b15-ehp0114-001348] Grimvall E, Rylander L, Nilsson-Ehle P, Nilsson U, Stromberg U, Hagmar L (1997). Monitoring of polychlorinated biphenyls in human blood plasma: methodological developments and influence of age, lactation, and fish consumption. Arch Environ Contam Toxicol.

[b16-ehp0114-001348] Groome NP, Illingworth PJ, O’Brien M, Pai R, Rodger FE, Mather JP (1996). Measurement of dimeric inhibin B throughout the human menstrual cycle. J Clin Endocrinol Metab.

[b17-ehp0114-001348] Guillette LJ, Brock JW, Rooney AA, Woodward AR (1999). Serum concentrations of various environmental contaminants and their relationship to sex steroid concentrations and phallus size in juvenile American alligators. Arch Environ Contam Toxicol.

[b18-ehp0114-001348] Hagmar L, Björk J, Sjödin A, Erfurth E-M (2001). Plasma levels of persistent organohalogens and hormone levels in adult male humans. Arch Environ Health.

[b19-ehp0114-001348] Henriksen GL, Michalek JE, Swaby JA, Rahe AJ (1996). Serum dioxin, testosterone, and gonadotropins in veterans of Operation Ranch Hand. Epidemiology.

[b20-ehp0114-001348] INUENDO 2006. European Commission R&D Project: Human Fertility at Risk from Biopersistent Organochlorines in the Environment? Available: http://www.inuendo.dk [accessed 17 July 2006].

[b21-ehp0114-001348] Johnson E, Shorter C, Bestervelt L, Patterson D, Needham L, Piper W (2001). Serum hormone levels in humans with low serum concentrations of 2,3,7,8-TCDD. Toxicol Ind Health.

[b22-ehp0114-001348] Jönsson BAG Rylander L Lindh C Rignell-Hydbom A Giwercman A Toft G 2005 Inter-population variations in concentrations, determinants of and correlations between 2,2′,4,4′,5,5′-hexachlorobiphenyl (CB-153) and 1,1-dichloro-2,2-bis (*p*-chlorophenyl)-ethylene (*p,p*′-DDE): a cross-sectional study of 3161 men and women from Inuit and European populations Environ Health 4 27 10.1186/1476-069X-4-27 [Online 11 November 2005]. 16283941 PMC1308838

[b23-ehp0114-001348] Kelce WR, Stone CR, Laws SC, Gray LE, Kemppainen JA, Wilson EM (1995). Persistent DDT metabolite, *p,p*′DDE is a potent androgen receptor antagonist. Nature.

[b24-ehp0114-001348] Kizu R, Okamura K, Toriba A, Kakishima H, Mizokami A, Burnstein KL (2003). A role of aryl hydrocarbon receptor in the antiandrogenic effects of polycyclic aromatic hydrocarbons in LNCaP human prostate carcinoma cells. Arch Toxicol.

[b25-ehp0114-001348] Longnecker MP, Rogan WJ, Lucier G (1997). The human health effects of DDT (dichlorodiphenyltrichloroethane) and PCBS (polychlorinated biphenyls) and an overview of organochlorines in public health. Annu Rev Public Health.

[b26-ehp0114-001348] Martin SA, Harlow SD, Sowers MF, Longnecker MP, Garabrant D, Shore DL (2002). DDT metabolite and androgens in African-American farmers. Epidemiology.

[b27-ehp0114-001348] Persky V, Turyk M, Anderson HA, Hanrahan LP, Falk C, Steenport DN (2001). The effects of PCB exposure and fish consumption on endogenous hormones. Environ Health Perspect.

[b28-ehp0114-001348] Pocar P, Fischer B, Klonisch T, Hombach-Klonisch S (2005). Molecular interactions of the aryl hydrocarbon receptor and its biological and toxicological relevance for reproduction. Reproduction.

[b29-ehp0114-001348] Richthoff J, Rylander L, Jönsson BA-G, Åkesson H, Hagmar L, Nilsson-Ehle P (2003). Serum levels of 2,2′,4,4′,5,5′-hexachlorobiphenyl (CB-153) in relation to markers of reproductive function in young males from the general Swedish population. Environ Health Perspect.

[b30-ehp0114-001348] Rignell-Hydbom A, Rylander L, Giwercman A, Jönsson BAG, Nilsson-Ehle P, Hagmar L (2004). Exposure to CB-153 and *p,p*′-DDE and male reproductive function. Hum Reprod.

[b31-ehp0114-001348] Sjödin A, Hagmar L, Klasson-Wehler E, Björk J, Bergman A (2002). Influence of the consumption of fatty Baltic Sea fish on plasma levels of halogenated environmental contaminants in Latvian and Swedish men. Environ Health Perspect.

[b32-ehp0114-001348] Skakkebæk NE, Rajpert-De Meyts E, Main KM (2001). Testicular dysgenesis syndrome: an increasingly common developmental disorder with environmental aspects. Hum Reprod.

[b33-ehp0114-001348] Spano M, Toft G, Hagmar L, Eleuteri P, Rescia M, Rignell-Hydbom A (2005). Exposure to PCB and *p,p*′-DDE in European and Inuit populations: impact on human sperm chromatin integrity. Hum Reprod.

[b34-ehp0114-001348] Svensson BG, Nilsson A, Jonsson E, Schutz A, Akesson B, Hagmar L (1995). Fish consumption and exposure to persistent organochlorine compounds, mercury, selenium and methylamines among Swedish fishermen. Scand J Work Environ Health.

[b35-ehp0114-001348] Tiido T, Rignell-Hydbom A, Jönsson BAG, Giwercman YL, Pedersen HS, Wojtyniak B (2006). Impact of PCB and *p,p*-DDE contaminants on human sperm Y:X chromosome ratio: studies in three European populations and the Inuit population in Greenland. Environ Health Perspect.

[b36-ehp0114-001348] Toft G Axmon A Giwercman A Thulstrup AM Rignell-Hydbom A Pedersen HS 2005 Fertility in four regions spanning large contrasts in serum levels of widespread persistent organochlorines: a cross-sectional study Environ Health 4 26 10.1186/1476-069X-4-26 [Online 9November 2005]. 16280075 PMC1308837

[b37-ehp0114-001348] Toft G, Rignell-Hydbom A, Tyrkiel E, Shvets M, Giwercman A, Lindh CH (2006). Semen quality and exposure to persistent organochlorine pollutants. Epidemiology.

[b38-ehp0114-001348] Toppari J, Larsen JC, Christiansen P, Giwercman A, Grandjean P, Guillette LJ (1996). Male reproductive health and environmental xenoestrogens. Environ Health Perspect.

[b39-ehp0114-001348] Vermeulen A, Verdonck L, Kaufman JM (1999). A critical evaluation of simple methods for the estimation of free testosterone in serum. J Clin Endocrinol Metab.

